# Grit as a moderator: the mediating role of self-efficacy and academic performance between conscientiousness and life satisfaction among college students

**DOI:** 10.3389/fpsyg.2025.1665636

**Published:** 2025-11-18

**Authors:** Jianan Li, Tingting Ma, Chang Seek Lee

**Affiliations:** 1 School of Digital Economics and Management, Guangzhou University of Software, Guangzhou, China; 2 Department of Lifelong Education, Hanseo University, Seosan, Republic of Korea

**Keywords:** academic performance, academic self-efficacy, conscientiousness, grit, life satisfaction, moderated mediation effect

## Abstract

**Introduction:**

In recent decades, academic research has established the significant influence of personality traits on life satisfaction. However, the mediating and moderating mechanisms underlying the relationship between conscientiousness and life satisfaction remain insufficiently understood, warranting further investigation.

**Methods:**

This study aimed to clarify the moderated mediation mechanism linking conscientiousness, academic self-efficacy, academic performance, grit, and life satisfaction. To this end, an exploratory hypothetical model was proposed, and empirical verification of the model was conducted via a random sampling survey. The respondents of this study were 304 undergraduate and professional students from China. The data analysis tools used in this study included SPSS AMOS Version.26, SPSS Statistics Ver.25 and SPSS PROCESS Macro-Ver.4.2.

**Results:**

The results showed that conscientiousness, academic self-efficacy, academic performance, life satisfaction and grit were positively correlated. Grit played a moderating role in the dual mediating relationship from conscientiousness to academic self-efficacy to academic performance to life satisfaction.

**Discussion:**

The research results provided new ideas for explaining the formation mechanism of college students’ life satisfaction, and provided more targeted suggestions on using grit to intervene and improve students’ happiness in school.

## 1 Introduction

As an important construct of positive psychology, life satisfaction (LS) has been regarded as a stable indicator of personal well-being (Gilman et al., 2000) and psychological development (Goldbeck et al., 2007) in adolescence. Against this backdrop of growing attention to understanding the predictors of adolescent LS, research on personality—particularly within the framework of the “Big Five Personality Model”—has emerged as a key avenue for exploring what shapes such life satisfaction. With the in-depth study of the “Big Five Personality Model,” conscientiousness has been repeatedly confirmed to be an important contributing factor to life satisfaction (Lounsbury et al., 2005). At the same time, other concepts related to cognitive psychology, such as academic self-efficacy (ASE) and academic performance (AP), had also been found to be used to further explain LS (Bradley and Corwyn, 2004; Van Petegem et al., 2007). However, to date, there was still a lack of sufficient research to explain the relationship between conscientiousness, ASE, AP, and LS within the framework of a moderated mediation model. The unclear causal relationship between these important variables would further hinder the development and application of effective educational interventions. Thus, clarifying the relationships among these variables is imperative. This study seeks to explore and explicate the moderated mediation effect between life satisfaction and its key influencing factors.

Academic self-efficacy beliefs touched virtually every aspect of students’ lives—whether they thought productively, pessimistically or optimistically, which suggested that students would choose to engage in activities in which they felt competent and avoided those in which they did not (Pajares, 2001). ASE had been shown to be related to conscientiousness (Lee and Klein, 2002) and LS (Pinquart et al., 2004). Besides, one of the most important learning outcomes for students (Dogan, 2015), academic performance, which referred to the level of success a student achieved in their educational pursuits (York et al., 2015), also presented the same characteristics as ASE’s connection with conscientiousness and LS. It had been shown that more conscientious students usually achieved better academic performance (Chamorro-Premuzic and Furnham, 2003; Heaven et al., 2007), which in turn lead to higher life satisfaction (Van Petegem et al., 2007). In addition, the causal relationship between ASE and AP had been repeatedly confirmed to be strong (Richardson et al., 2012). Based on the above previous research results, ASE and AP were likely to play a mediating role in the causal relationship between conscientiousness and life satisfaction, either separately or jointly.

In addition, a large number of research results confirmed that grit differences had a significant impact on students’ academic performance levels, as grit could help students maintain perseverance and enthusiasm for long-term goals and controlled their emotions and desires in challenging or difficult situations (Kannangara et al., 2018). Specifically, it was verified that compared with students with lower levels of grit, students with high levels of grit were more likely to have self-confidence, proactively faced expected failures, overcame obstacles in the pursuit of academic and professional achievements, explored different tasks in future careers and academic pursuits, and encouraged themselves to adapt to society in the face of adversity (Kundu, 2017). Other studies had shown that grit can moderate the relationship between college students’ learning engagement and academic outcomes, which meant that students with higher levels of grit devoted more energy to the learning process and thus achieved higher academic outcomes (Hodge et al., 2017). In this sense, grit had the potential to moderate the dual mediating effects between conscientiousness, ASE, AP, and LS. However, to date, no research had confirmed such results, so this study aimed to fill this gap. In order to verify the intrinsic relationship between the above core variables, the research questions are set as follows: First, what are the interrelationships between conscientiousness, ASE, AP, and LS? Second, in the dual mediating effect of ASE and AP between conscientiousness and LS, does grit have a moderated mediating effect?

## 2 Literature review

### 2.1 Conscientiousness and life satisfaction

Conscientiousness was rooted in the Big Five personality theory, a construct that described individual differences in self-control, responsibility to others, diligence, orderliness, and rule-following (Roberts et al., 2009). Indeed, conscientiousness represented a person’s traits that included competence, order, responsibility, achievement-seeking, self-discipline, and thoughtfulness (McGhee et al., 2007), qualities that were consistent with academic orientation and subjective well-being. Contemporary research had assumed a three-component structure for subjective wellbeing: positive affect, negative affect, and life satisfaction (Andrews and Withey, 1976).

Life satisfaction was defined as the universal evaluation of an individual’s living standards according to the criteria that the individual determined (Shin and Johnson, 1978). As an important concept in positive psychology, life satisfaction has been regarded as a stable contributor of students’ well-being (Gilman et al., 2000) and psychological development (Goldbeck et al., 2007). During the past 20 years, the focus of studies on life satisfaction had shifted from exploring its measurement to analyzing its influential factors, suggesting that an individual’s life satisfaction was influenced by subjective variables, such as personality (James et al., 2012).

Personality and temperament variables had been demonstrated to account for most of the variance in subjective wellbeing (Emmons and Diener, 1985; Fogle et al., 2002). In a major work with a meta-analytic approach to test the viability of several top-down and bottom-up models, the findings indicated that conscientiousness was related to both life satisfaction and satisfaction within various specific domains (Heller et al., 2004). There was more evidence showing that conscientiousness was significantly related to life satisfaction among a Chinese undergraduate sample (Zhang and Zheng, 2005). Previous research found that stronger conscientiousness showed a consistent association with higher scores in life satisfaction (Baudin et al., 2011). Moreover, research results indicated that adolescents with higher levels of conscientiousness showed more satisfaction with their lives than adolescents with lower levels of conscientiousness (Lowenstein et al., 2007). More recently, it was tested in a set of equations regressed conscientiousness variable on life satisfaction that conscientiousness accounted for a significant amount of variance (11.1%) in life satisfaction (Jie and Du, 2015). However, the academic community has not reached a clear consensus on the mediating mechanism between conscientiousness and life satisfaction. Therefore, what mediates the relationship between these two variables needs further exploration.

### 2.2 Dual mediating effect of academic self-efficacy and academic performance

According to social cognitive theory, academic self-efficacy referred to learners’ judgment of their ability to achieve expected results in their academic field (Bandura, 1997). It was further explained that academic self-efficacy was the perception of a student about his/her abilities and aspects that directed him/her to the way of success (Sirois, 2004). More importantly, research results in recent decades had confirmed that ASE is positively correlated with both conscientiousness (Chen et al., 2001) and LS (Azar et al., 2006). Further research also showed that the more responsible students were, the better their self-learning efficiency (ASE) would be (Lee and Klein, 2002), and this improvement in ASE would eventually have a positive impact on the life satisfaction of these students (Suldo and Huebner, 2006). In other words, there were clear positive causal relationships between academic self-efficacy and conscientiousness and life satisfaction, and this connection laid a theoretical foundation for ASE to become a potential mediating variable connecting the above paths.

Next, academic achievement was one of the most important learning outcomes for students (Dogan, 2015). Academic achievement referred to the degree of success that students achieved in their educational pursuits (Kuh et al., 2006; York et al., 2015). In addition, studies had found a positive correlation between AP and conscientiousness, while neuroticism was negatively correlated with AP (Poropat, 2009). Other studies had also shown that higher life satisfaction (Rode et al., 2005) and higher well-being (Chow, 2007) were associated with higher GPA. In addition, the fact that conscientious students were able to achieve greater academic success had been repeatedly confirmed, both in high school (Trautwein et al., 2009) and in college (Chamorro-Premuzic and Furnham, 2008). A 6-year longitudinal study of adolescent development in Hong Kong found that students who had a more negative view of their school performance were more likely to be dissatisfied with life than those who had a more positive view of their school performance (Shek and Li, 2016). There was a strong causal relationship between AP, conscientiousness, and LS, which had been widely confirmed by the academic community. Therefore, academic performance may play a mediating role in the link between conscientiousness and life satisfaction.

In addition, ASE was also positively correlated with AP and had been repeatedly confirmed to be a significant predictor of student academic achievement (Chemers et al., 2001), which meant that the path from ASE to AP was also theoretically feasible. Therefore, based on the above theoretical basis for the causal relationship between conscientiousness, ASE, AP, and LS, the path from conscientiousness to LS via ASE and AP seemed logical and feasible. Previous studies had attempted to combine academic self-efficacy and life satisfaction within a complex model, specifically, a linear model was created considering that a happy school life was affected by general self-efficacy, academic self-efficacy and life satisfaction (Döş, 2023). However, research examining moderated dual mediation effects of the kind explored in this study remains relatively limited.

### 2.3 Moderating effect of grit

Grit was defined as working strenuously toward challenges, maintaining effort and interest despite failure, adversity, and plateaus in progress (Duckworth et al., 2007). In other words, people with greater levels of grit were more determined when trying to overcome obstacles (Arslan et al., 2013). More importantly, it was tested that there were significant correlations between grit and life satisfaction (Kim et al., 2022). And grit was also positively connected with academic performance, which was supported by a survey of 400 undergraduates from Malaysia (Siah et al., 2018).

More importantly, in educational psychology research, grit was often used as a moderating variable, and this had a solid research foundation. To name a few examples, research showed that grit strengthened the relationship between student adaptability and self-control, highlighting its role as a powerful, positive force that promoted sustained self-control, self-regulation, and self-motivation in the face of difficulty and setbacks (Ma et al., 2020). Also, grit was concluded to moderate the relationship students’ intention to participate in oral presentations and their actual oral presentation performance in university in Malaysia (Anuar et al., 2021). More recently, in a Korean study, a moderated mediation effect of grit on the path from organizational incivility to life satisfaction via job stress and gratitude was verified (Kim et al., 2022).

Overall, these findings highlighted the importance of considering grit as a moderating variable when examining life satisfaction, as it played an important role in enhancing students’ educational confidence and feelings. Based on previous research, it seemed likely that grit played a moderated dual mediating role in the path from conscientiousness via academic self-efficacy and academic performance to life satisfaction.

## 3 Research methodology

### 3.1 Research model

**Figure 1:**
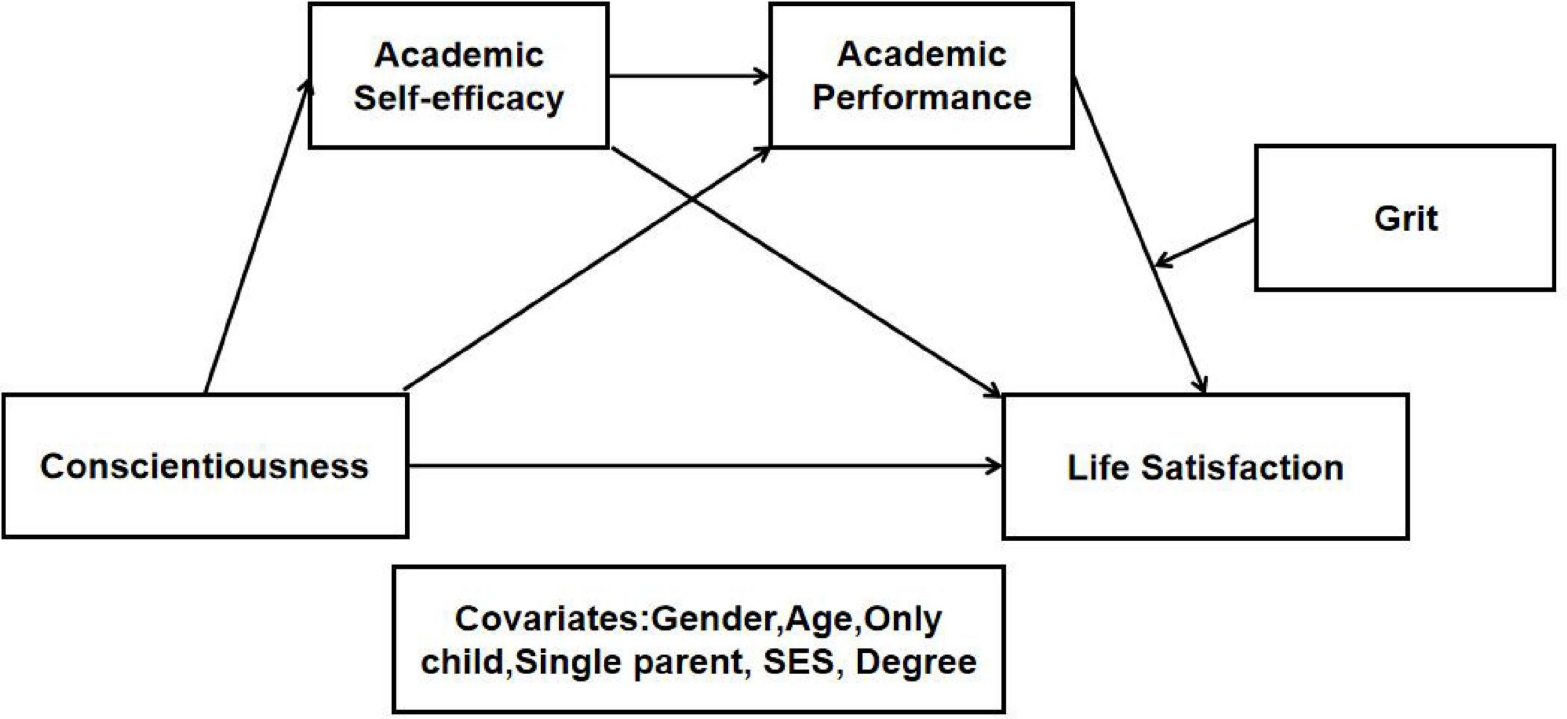
Proposed research model.

The hypothesized model proposed in this study was shown in Figure 1. This model was used to verify the dual mediating role of ASE and AP in the path from conscientiousness to LS, and further, to prove whether grit had a moderated dual mediating role in the above relationship. The covariates in the hypothesized model included: gender, age, only child, single parent, socioeconomic status (SES), and school level.

### 3.2 Respondents of the study

The respondents of this study were college students in China, and the sample had covered major provincial administrative regions in China (including provinces, autonomous regions, municipalities directly under the central government, and special administrative regions). A total of 304 college students were interviewed, and their overall characteristics were as follows: 40.1% were male and 59.9% were female. In terms of age, 12.5% of the respondents were under 19 years old, 30.6% were under 20 years old, 26.6% were under 21 years old, 22.7% were under 22 years old, and 7.6% were over 22 years old. Among them, 41.4% of the respondents were not only children, and 58.6% of the respondents were only children. In addition, 8.2% of the respondents came from single-parent families, and 91.8% of the respondents were from two-parent families. In terms of socioeconomic status, 25.7% of the respondents came from wealthy families, 53.6% of the respondents came from ordinary families, and 20.7% of the respondents came from middle-class families. In terms of the current educational background of the respondents, 49.7% of the respondents were four-year undergraduates, and 50.3% of the respondents were three-year college students.

### 3.3 Ethical statement

This study was conducted in strict accordance with the Ethical Guidelines for Psychological Research Involving Human Subjects (promulgated by the Chinese Psychological Society) and the core principles of the Declaration of Helsinki. Given the non-invasive nature of the research—specifically, data were collected solely through anonymous online questionnaires, focusing on non-sensitive demographic information (e.g., gender, age) and self-reported psychological scale responses (without collecting personal identifiers such as names, student IDs, or contact details)—the study did not involve risks to participants’ physical or mental health, privacy, or legitimate rights.

### 3.4 Data gathering procedures

The two-week sampling work started on June 17, 2024, and the sampling subjects were selected from 870,000 Chinese college students registered on the Wenjuanxing platform. The sampling method was simple random sampling. First, 3,782 college students registered on the platform were randomly selected as invitation subjects through the system software. Then, the research team sent an invitation letter (including informed consent and questionnaire) to these selected students. The invitation letter explained in detail the purpose, process, benefits, risks, confidentiality, voluntary participation and withdrawal terms of this study. Only students who confirmed the informed consent would be able to enter the formal sampling procedure. In the end, 1,323 college students had confirmed the consent and participated in the study. In order to reduce common method bias, participants were asked to answer the questionnaire anonymously, and some items were reverse scored. Subsequently, the research subjects were asked to complete the online questionnaire through the “Wenjuanxing” platform, and 1,040 participants submitted the questionnaire results. Participants who answered and submitted the questionnaire in full will receive a reward of 5 yuan (a non-coercive incentive common in Chinese college student surveys). Next, the research team verified the 1,040 submitted questionnaires to ensure data quality, eliminating 737 invalid ones based on three key criteria: (1) incomplete responses; (2) inattentive completion (a threshold set via pre-survey); (3) logical contradictions (e.g., conflicting answers to consistent conscientiousness items) or uniform Likert-scale responses. Finally, 304 results were marked as valid questionnaires, and their data were used for subsequent analysis.

### 3.5 Research instrument

#### 3.5.1 Conscientiousness

The conscientiousness scale was originated from Costa and McCrae’s NEO-PI-R (Costa and McCrae, 2000). The brief version of Chinese Big Five Personality Inventory (CBF-PI-B) developed by Wang et al. (2011) was used and modified by the researcher in this study. This modified sub-scale of conscientiousness consisted of 4 items. It was a 6-point Likert scale ranging from 1 point of “Not at all consistent” to 6 points of “Completely consistent.” Lower points indicated a weaker level of conscientiousness. Question 4 was rhetorical. The questions included: “Once I have set a goal, I will persist and work hard to achieve it.”; “I work or study very hard.”; “I am a person who gives my best to do things.” and “At work, I often just want to get by.” This modified scale was verified to have good validity and various indicators were used for assessment at an item level (PCMIN/df = 2.773, CFI = 0.991, TLI = 0.991, AGFI = 0.953, RMSEA = 0.076, KMO = 0.780, Total Variance Explained = 63.52%). There were no cross loadings between factors. The reliability of conscientiousness was high (Cronbach’s *α* = 0.799).

#### 3.5.2 Academic self-efficacy

The academic self-efficacy scale used in this study was originally developed by Midgley (2000) and modified by Chen et al. (2016) for the Chinese situation. This scale was adjusted for better use by the research. This scale was modified into 4 items. It was a 5-point Likert scale ranging from 1 point of “strongly disagree” to 5 points of “strongly agree.” The higher the score, the higher the level of ASE. The questions were: “I’m certain I can master the skills taught in class this year”; “I’m certain I can figure out how to do the most difficult class work”; “Even if the work is hard, I can learn it”; “I can do even the hardest work in this class if I try.” This revised scale was of good validity (PCMIN/df = 2.601, CFI = 0.990, TLI = 0.992, AGFI = 0.958, RMSEA = 0.073, KMO = 0.774, Total Variance Explained = 60.45%). The reliability of ASE was high (Cronbach’s *α* = 0.777).

#### 3.5.3 Academic performance

To measure the academic performance of college students in China, the scale developed by Li and Lee (2023) was used in this study. It was a self-reported scale and had a total of 4 items. The questions included “My school professional curriculum has excellent grades”; “My school’s elective courses have excellent grades”; “My school’s public curriculum has excellent grades, such as: higher mathematics, ideological and moral cultivation, sports, etc.”; “My school’s other courses have excellent grades.” It was a 5-point Likert scale ranging from 1 point of “strongly disagree” to 5 points of “strongly agree.” The higher the score, the higher the level of AP. This scale was verified to have good validity (PCMIN/df = 2.615, CFI = 0.987, TLI = 0.992, AGFI = 0.959, RMSEA = 0.073, KMO = 0.752, Total Variance Explained = 56.02%). This study’s reliability of AP was acceptable (Cronbach’s *α* = 0.738).

#### 3.5.4 Grit

To measure the grit level of Chinese college students, the scale originally developed by Duckworth and Quinn (2009) and modified for Chinese situation by Xie et al. (2017) was taken in this research. It was a 5-point Likert scale ranging from 1 point of “strongly disagree” to 5 points of “strongly agree.” The higher the score, the higher the level of grit. Originally, this scale was of two subscales and had a total of 12 items. The CI and PE subscales were comprised of scores from questions (2, 3, 5, 7, 8, 11) and (1, 4, 6, 9, 10, 12) respectively. However, this scale was modified by the researcher for better validity in this study. More specifically, the scale now excluded question number 2 and number 10, which were: “New ideas and projects sometimes distract me from previous ones” and “I have achieved a goal that took years of work.” The revised scale was verified to have better validity (PCMIN/df = 1.867, CFI = 0.975, TLI = 0.961, AGFI = 0.933, RMSEA = 0.053, KMO = 0.875, Total Variance Explained = 58.84%). This study’s reliability of grit scale was high (Cronbach’s *α* = 0.853). The reliability for the subscales CI (Cronbach’s α = 0.816) and PE (Cronbach’s α = 0.811) were also high.

#### 3.5.5 Life satisfaction

To measure the level of life satisfaction of Chinese college students, the Satisfaction with Life Scale (SWLS) scale originally developed by Diener et al. (1985) and modified for Chinese situation by Shao (1993) was taken in this research. This five-item instrument was rated on a seven-point Likert scale, ranging from one point of very strongly disagree to seven points of very strongly agree, where a higher score indicated higher life satisfaction. It had a total of 5 items. There were questions like “So far I have gotten the important things I want in life” and “If I could live my life over, I would change almost nothing.” This scale was verified to have good validity (PCMIN/df = 1.365, CFI = 0.998, TLI = 0.993, AGFI = 0.973, RMSEA = 0.035, KMO = 0.873, Total Variance Explained = 69.26%). The reliability of LS scale in this study was good (Cronbach’s *α* = 0.885).

#### 3.5.6 Covariates

Other personal characteristics that affect the mediating variables and dependent variables were set as control variables during the analysis, including: gender, age, only child, single parent, socioeconomic status, and school level.

### 3.6 Statistical tools (for data analysis)

In the process of data analysis, the analysis tools used included SPSS AMOS Version.26, SPSS Statistics Ver.25 and SPSS PROCESS Macro-Ver.4.2. More specifically, frequency analysis was employed to understand and illustrate the main characteristics of the participants. Next, exploratory factor analysis and confirmatory factor analysis were applied to modified and guarantee the validity of all the scales. Then, the reverse question was addressed and Cronbach’s α was calculated to ensure the reliability of the variables. Subsequently, to find the answer to the first research question, the Pearson bivariate correlation method was applied to demonstrate the relationships between the core variables. Finally, for the second research question, SPSS PROCESS Macro Model No. 87 was used to present the moderated dual mediation paths between the variables. In addition, to analyze the moderated mediation effect, prior to the analysis, conscientiousness and grit were mean-centered, the confidence level of the output confidence interval was 95%, and the number of bootstrap samples for the percentile bootstrap confidence interval was 5,000.

## 4 Results

### 4.1 Correlation between main variables

The correlations within the main variables were presented in Table 1 through Pearson correlation analysis. Specifically, conscientiousness positively correlated with ASE (*r* = 0.630 *p* < 0.001), AP (*r* = 0.585, *p* < 0.001), grit (*r* = 0.722, *p* < 0.001) and LS (*r* = 0.459, *p* < 0.001). ASE positively correlated with AP (*r* = 0.615, *p* < 0.001), grit (*r* = 0.637, *p* < 0.001) and LS (*r* = 0.530, *p* < 0.001). AP positively correlated with grit (*r* = 0.538, *p* < 0.001) and LS (*r* = 0.501, *p* < 0.001). Grit positively correlated with LS (*r* = 0.516, *p* < 0.001). Notably, the correlation coefficient between conscientiousness and grit was larger than 0.7, suggesting multicollinearity problem. Accordingly, regression analysis was conducted to calculate the VIF and tolerance values, with LS as the dependent variable and grit as independent variables. The results showed that the tolerance value of the independent variable was 1, and the VIF value of the model was 1. According to Belsley (1980), a tolerance value below 0.10 or a VIF above 10 implies multi-collinearity, so there was no problem of multicollinearity for the current results. Furthermore, through frequency analysis, the value of conscientiousness averaged at 4.57 (range 1 to 6), ASE averaged at 3.57 (range 1 to 5), AP averaged at 3.84 (range 1 to 5), grit averaged at 3.38 (range 1 to 5), and LS averaged at 4.28 (range 1 to 7). Additionally, the skewness and kurtosis indices showed a normal distribution for all variables considered in the present study (values between −1 and 1) (Aluja et al., 2007).

**Table 1:** Results of correlation and descriptive statistics analysis.

Variable	1	2	3	4	5
1. Conscientiousness	1				
2. ASE	0.630^***^	1			
3. AP	0.585^***^	0.615^***^	1		
4. Grit	0.722^***^	0.637^***^	0.538^***^	1	
5. LS	0.459^***^	0.530^***^	0.501^***^	0.516^***^	1
M	4.5740	3.5650	3.8413	3.3809	4.2842
SD	0.81894	0.71414	4.63462	0.67557	1.33142
Skewness	−0.655	−0.440	−0.713	−0.218	−0.248
Kurtosis	0.130	−0.377	0.247	−0.642	−0.802

***p < 0.001; ASE, Academic Self-efficacy; AP, Academic Performance; LS, Life Satisfaction.

### 4.2 Moderated mediation effect

**Figure 2:**
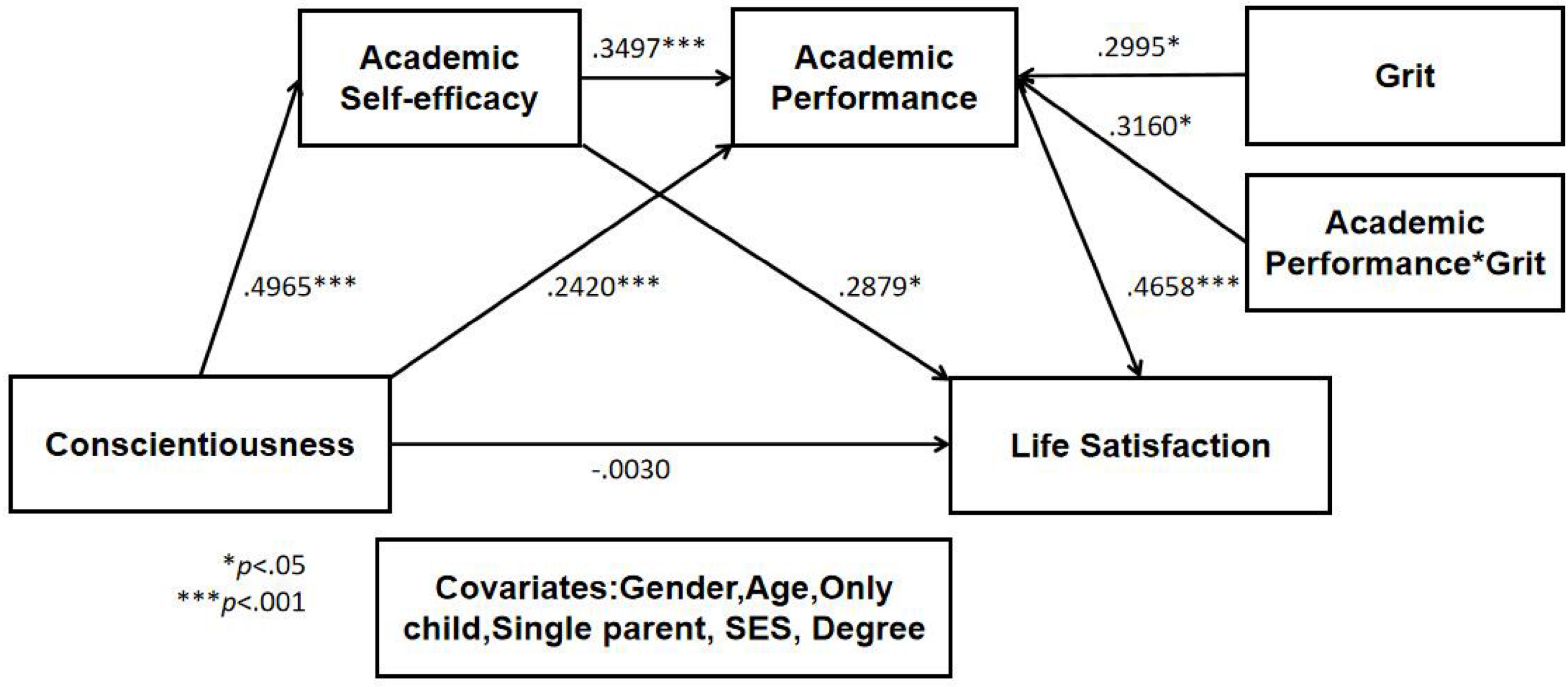
Statistical model of moderated mediation effect.

**Figure 3:**
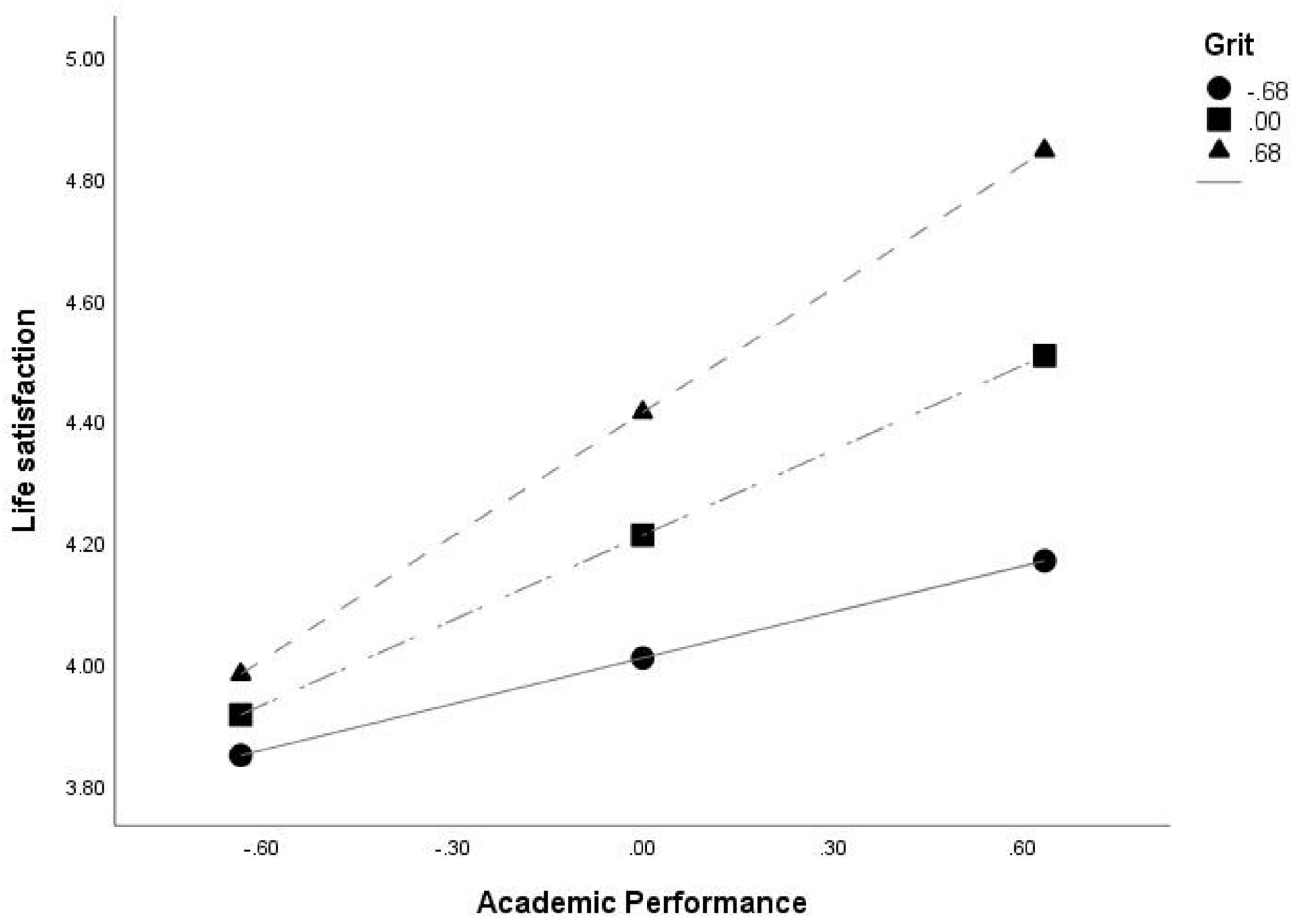
Moderating effect of grit on the relationship between AP and LS under Model 87.

The moderated mediation effect of grit on the path from conscientiousness to LS through ASE and AP was verified by model No.87 of SPSS PROCESS macro. The results were displayed in Tables 2, 3 and Figures 2, 3.

**Table 2:** Results of moderated mediation effect analysis.

Variables		Mediating variable model 1 (DV: ASE)			Mediating variable model 2 (DV: AP)			Dependent variable model (DV: LS)		
		Coeffect	SE	*t*-value	Coeffect	SE	*t*-value	Coeffect	SE	*t*-value
Constant		1.9860	0.3696	5.3732^***^	−2.3743	0.3384	−7.0158^***^	3.9610	0.7952	4.9813^***^
ID	Conscientiousness	0.4965	0.0405	12.2608^***^	0.2420	0.0435	5.5675^***^	−0.0030	0.1058	−0.0281
M1	ASE				0.3497	0.0508	6.8841^***^	0.2879	0.1133	2.5411^*^
M2	AP							0.4658	0.1234	3.7742^***^
W	Grit							0.2995	0.1270	2.3587^*^
Int	AP*Grit							0.3160	0.1397	2.2629^*^
Highest order test	R^2^ change							0.5137		
	F							28.0396^***^		
Covariates	Gender	−0.1349	0.0656	−2.0568^*^	0.0807	0.0577	1.3975	0.1032	0.1176	0.8780
	Age	0.0311	0.0283	1.1019	0.0067	0.0247	0.2721	0.0870	0.0498	1.7487
	Only child	−0.0371	0.0668	−0.5558	0.0218	0.0584	0.3725	−0.1022	0.1171	−0.8728
	Single parent	−0.0184	0.1173	−0.1566	−0.0395	0.1025	−0.3858	0.5002	0.2061	2.4264^*^
	SES	−0.1473	0.0432	−3.4087^***^	−0.0879	0.0385	−2.2831^*^	−0.6249	0.0784	7.9674^***^
	Degree	−0.0197	0.0642	−0.3069	0.1177	0.0561	2.0994^*^	−0.0676	0.1133	−0.5971
Model Summary	R^2^	0.4408			0.4609			0.0085		
	F	33.3366^***^			31.5292^***^			5.1205^*^		

Conditional effects of grit					
Grit	Effect	BOOT SE	T value	^*^LLCI	^**^ULCI
−0.6756 (M−SD)	0.2523	0.1292	1.9525	−0.0020	0.5067
0.0000 (M)	0.4658	0.1234	3.7742^***^	0.2229	0.7088
0.6756 (M + SD)	0.6794	0.1777	3.8233^***^	0.3296	1.0291

Moderator value(s) defining Johnson-Neyman significance region(s)		
Value	% below	% above
−0.6709	17.4342	82.5658

Conditional effect of focal predictor at values of the moderator					
Grit	Effect	BOOT SE	T value	^*^LLCI	^**^ULCI
−1.6809	−0.0654	0.2277	−0.2872	−0.5135	0.3828
⋮					
−0.7209	0.2380	0.1321	1.8023	−0.0219	0.4979
−0.6709	0.2538	0.1290	1.9681	0	0.5076
−0.5609	0.2886	0.1233	2.3407^*^	0.0459	0.5312
⋮					
1.5191	0.9459	0.2774	3.4097^***^	0.3999	1.4919

^*^p < 0.05, ^**^p < 0.01, ^***^p < 0.001, ID, Independent variable; DV, Dependent variable; M, Mediating variable; W, Moderating variable; ASE, Academic Self-efficacy; AP, Academic Performance; LS, Life Satisfaction; Int, Interaction terms of variables; ^*^LLCI = 95% lower level of confidence intervals, ^**^ULCI = 95% upper level of confidence intervals.

**Table 3:** Analysis of direct and indirect effects.

Direct effect (Conscientiousness-Life Satisfaction)				
Effect	BOOT SE	T value	^*^LLCI	^**^ULCI
−0.0030	0.1058	−0.0281	−0.2111	0.2052

Total indirect effect			
Indirect effect (Conscientiousness-Academic Self-Efficacy-Life Satisfaction)			
Effect	BOOT SE	^*^LLCI	^**^ULCI
0.1430	0.0656	0.0144	0.2750

Conditional indirect effects (Conscientiousness-Academic Performance-Life Satisfaction)				
Grit	Effect	BOOT SE	^*^LLCI	^**^ULCI
−0.6756 (M−SD)	0.0611	0.0334	0.0040	0.1360
0.0000 (M)	0.1127	0.0401	0.0435	0.1998
0.6756 (M + SD)	0.1644	0.0576	0.0620	0.2890

Index of moderation mediation				
Grit	Index	BOOT SE	^*^LLCI	^**^ULCI
	0.0765	0.0367	0.0055	0.1524

Moderation mediating effect (Conscientiousness-Academic Self-Efficacy-Academic Motivation-Academic Performance)				
Grit	Effect	BOOT SE	^*^LLCI	^**^ULCI
−0.6756 (M−SD)	0.0438	0.0234	0.0029	0.0956
0.0000 (M)	0.0809	0.0269	0.0334	0.1384
0.6756 (M + SD)	0.1180	0.0384	0.0485	0.1993

Index of moderation mediation				
Grit	Index	BOOT SE	^*^LLCI	^**^ULCI
	0.0549	0.0251	0.0042	0.1047

*LLCI = 95% lower level of confidence intervals.

**ULCI = 95% upper level of confidence intervals.

Effect sizes were interpreted per Cohen’s (1988) criteria: 0.01 (trivial), 0.20 (small), 0.50 (medium), 0.80 (large). 95% CIs excluding zero indicate statistically significant effects (Hayes, 2018).

First of all, conscientiousness positively influenced ASE (0.4965, *p* < 0.001) and AP (0.2420, *p* < 0.001). Also, ASE positively affected AP (0.3497, *p* < 0.001) and LS (0.2879, *p* < 0.05). And lastly, AP significantly influenced LS in a positive way (0.4658, *p* < 0.001).

Secondly, the interaction of conscientiousness and grit positively influenced LS (0.3160, *p* < 0.05), and the R^2^ increased according to the interaction term between AP and grit (∆R^2^ = 0.5137, *p* < 0.001) was also significant, indicating a moderating effect. In order to confirm the pattern of interaction, the change was analyzed by dividing grit into three conditions: M-SD, M and M + SD. And the conditional effects of AP on LS according to the value of grit was found to be significant for college students with the average and higher level of grit (*p* < 0.001). These findings suggested that as grit increased, the effect of AP on LS was strengthened.

To identify the specific range of grit values where the effect of AP on LS remained significant, the Johnson-Neyman technique was used for analysis. The conditional effect of AP was statistically significant when grit was above B = −0.6709 (covering 82.57% of the sample). This indicated that AP as a stronger predictive factor under conditions of average or higher levels of grit, but its impact diminished when grit was lower.

More specifically, the moderating effect of grit between AP and LS was also shown in Figure 3. When grit was not high, the slope of increasing LS as AP increased was relatively gentle, but when grit was higher, the slope of increasing LS as AP increased was relatively steep. In other words, as academic performance increased, life satisfaction also increased, but the rate of increase was greater among college students with higher level of grit.

Lastly, in order to verify the moderated dual mediation effect, the conditional and non-conditional indirect effect of grit was analyzed, and the results were displayed in Table 3. The direct effect of conscientiousness on LS was no longer significant, as zero did exist in 95% of the confidence interval (−0.2111 to 0.2052). Moreover, the indirect effect from conscientiousness to academic self-efficacy to life satisfaction was significant, as zero did not exist in 95% of the confidence interval (0.1444 to 0.2750). Further, the conditional indirect effect of AP in the relationship between conscientiousness and LS was also significant in all three conditions of grit, as zero did not exist in 95% of the three confidence intervals of grit, respectively. More importantly, in the effect of conscientiousness on LS, the conditional indirect effect of ASE and AP was significant in all conditions [M−SD (0.0029 to 0.0956), M (0.0334 to 0.1384) and M + SD (0.0485 to 0.1993)] of grit, which indicated a wider range of adaptability of grit’s moderating effect when ASE and AP were in together included in the whole model. The adjusted index of moderated mediation of grit was 0.0549, which was significant because there was no zero in the 95% confidence interval (0.0042 to 0.1047). Given the results above, the moderated complete dual mediating effect of grit was verified in the path from conscientiousness to LS through ASE and AP. Notably, the effect sizes of the key paths reflected their practical significance: the moderated mediation index of grit was 0.0549 (95% CI [0.0042, 0.1047], no zero included), indicating a small-to-medium practical effect (Cohen, 1988). For the dual mediating path (Conscientiousness→ASE → AP → LS), the indirect effect size increased from 0.0438 (95% CI [0.0029, 0.0956]) at grit’s M-SD to 0.1180 (95% CI [0.0485, 0.1993]) at grit’s M + SD, and for the path (Conscientiousness→AP → LS), the effect size rose from 0.0611 (95% CI [0.0040, 0.1360]) to 0.1644 (95% CI [0.0620, 0.2890]). All 95% CIs exclude zero, confirming the robustness of the moderated mediation effect.

## 5 Discussion

The above findings indicated that there were correlations among the core discussed variables and that there was a moderated mediation effect. These findings were further discussed below.

First, regarding the correlation between the core variables, the results of this study were consistent with those of the previous studies, and there was an expected strong positive correlation between the main variables. Specifically, consistent with previous research results, life satisfaction was positively correlated with conscientiousness (Heller et al., 2004), academic self-efficacy (Azar et al., 2006), and academic performance (Rode et al., 2005). In addition, academic self-efficacy and academic performance were positively correlated (McGeown et al., 2014), and they were also positively correlated with conscientiousness (Chemers et al., 2001). Through the comparison of the above research results, it was found that the positive correlation between the core variables studied in this paper reflected consistency and stability. This also showed that it was feasible to use the correlation between the above variables as the research basis for similar research hypotheses.

Secondly, consistent with the predictions of the previous studies, grit moderated the path from academic performance to life satisfaction, and different levels of grit had significant regulatory differences on students (Ma et al., 2020; Anuar et al., 2021). Specifically, similar to previous research results that higher levels of grit would bring about a reinforcing effect (Kim et al., 2022), this study found that the life satisfaction of college students with high levels of grit increased rapidly as their academic performance improved. This research phenomenon showed that grit, as a special quality, actually played a very powerful role in the intervention measures for students’ life satisfaction, which can be fully utilized in future intervention activities.

Finally, as expected by the theoretical logic, the dual mediation effect of ASE and AP was successfully verified, and the moderated dual mediation effect was also confirmed. Specifically, the significant indirect effect of ASE between conscientiousness and LS was verified, which was consistent with the causal logic that high conscientiousness leads to high ASE (Lee and Klein, 2002), and high ASE increased LS in the previous study (Suldo and Huebner, 2006). However, this result was inconsistent with a Canadian sample consisted of 71 participants, which found that academic satisfaction and school connectedness accounted for 49% of the variance in satisfaction with life, and that academic self-efficacy and college gratitude were not significant predictors of satisfaction with life (Nordstokke, 2019). This inconsistency could primarily stemmed from differences in sample characteristics (theirs was a small sample of Canadian first-year university students, while ours was a large sample of Chinese college students across multiple grades), cultural contexts (individualism vs. collectivism), and analytical models (linear regression for direct prediction vs. a moderated mediation model for indirect paths). These different research results also suggested that more relevant empirical research was needed in the future to verify the situation in different countries. In addition, the mediating effect of AP was also verified. In other words, AP explained the path from conscientiousness to LS, which was also consistent with the causal logic reflected in previous studies (Trautwein et al., 2009; Chamorro-Premuzic and Furnham, 2008; Shek and Li, 2016), and this effect still held under the moderation of different levels of grit. This finding indicated that academic self-efficacy and academic performance as well as grit played an important role in the path from conscientiousness to students’ life satisfaction. These results did not rule out the possibility that other mediating variables (such as academic motivation or growth mindset) played a role between conscientiousness and LS, which needed to be further confirmed by future research. More importantly, under the joint dual mediation effect of ASE and AP between conscientiousness and LS, grit still moderated this dual mediation relationship, in which high grit levels played a reinforcing role. This showed that the relationship between conscientiousness, academic self-efficacy, and academic performance jointly explained how they affected students’ life satisfaction, and they played a role individually. Grit had a significant impact on the path from the independent variable to the mediating variable in the entire model. The results of this study are unique to date and have pioneering significance in the comprehensive use of multiple psychological variables for positive psychology. At the same time, it also pays attention to the significance of the moderating effect of differences in college students’ grit levels. The practical value reflected in the results of the above studies can be used for more targeted theoretical research or more practical intervention project design in the future.

## 6 Conclusion

In summary, this random sampling study proved that the level of grit moderated the dual mediation relationship in the dual mediation path of academic self-efficacy and academic performance from conscientiousness to life satisfaction.

As for the limitations of this study, first, the college student sample used in this paper was limited to one country, and the information it can represent was still relatively limited. In the future, more research was needed on the relevant situation of college students in more other countries or regions in order to had a more comprehensive understanding of the current situation worldwide and the consistency and stability of this theory. Secondly, grit also included two sub-dimensions (perseverance of effort and consistency of interests), which this paper failed to further analyze. Finally, this paper did not decide to use longitudinal research for comparative analysis, which was limited in the reliability of comparison.

Overall, this paper is the only article that has been found so far that uses a simple random sampling method to verify that grit has a moderated dual mediation effect in the relationship between conscientiousness, academic self-efficacy, academic performance and life satisfaction. The research results of this paper provide new perspectives and inspiration for research in specific areas of this discipline, and also lay an empirical foundation for further research in the future.

As for the relevant suggestions of this article, with the deepening of understanding and research on the factors affecting students’ life satisfaction and their interrelationships and moderating variables, the following educational measures related to positive psychological education can be tried and carried out. Specifically, first of all, “conscientious behavior-efficacy” linkage training can be designed to strengthen the initiation of the basic path. For example, a “structured task list” can be embedded in professional courses, specific behavioral indicators of conscientiousness can be clarified, and the degree of behavioral completion can be recorded through an online platform. Starting with the independent variable “conscientiousness,” through specific behavioral training and immediate feedback, students can intuitively perceive the role of conscientiousness in promoting academic self-efficacy and activate the starting point of the mediating path. Second, teachers could construct the ladder tasks of grit empowerment and performance transformation to amplify the regulatory effect. In general courses or professional core courses, set up “three-level challenge tasks” (basic tasks-advanced tasks-innovative tasks), requiring students to complete them progressively for 3 consecutive weeks (cultivating the “persistence” of grit). Educators can cultivate grit through step-by-step tasks, and at the same time make the transformation process from academic performance to life satisfaction explicit, and use the regulatory effect of grit to amplify the effect of this path. Third, a full-cycle support closed loop can be established to integrate dual mediators and moderating variables. In other words, through cross-scenario integrated training, the dual-mediator path is activated synchronously, and with the help of the moderating effect of grit, the growth in the academic field can be more efficiently transformed into life satisfaction. The above suggestions transform abstract psychological mechanisms into perceptible and operational educational practices for college students through specific task design, feedback mechanism and scenario integration. From this perspective, the research results of this article will play a key role in future educational optimization practices, so that students can truly improve their academic performance and life satisfaction.

For future research directions, two key avenues are recommended: First, larger-scale longitudinal studies are warranted to validate and refine the current findings, as such designs can better capture the dynamic interplay of variables over time and enhance the generalizability of conclusions. Second, expanding the proposed model to incorporate more context-specific moderators (e.g., institutional support mechanisms) and mediators (e.g., academic resilience) would help elaborate the theoretical framework, providing a more comprehensive understanding of pathways for intervening in college students’ life satisfaction and academic development.

## Data Availability

The raw data supporting the conclusions of this article will be made available by the authors, without undue reservation.

## Author’s note

The author did not preregister this research in an independent, institutional registry.
